# The zinc-finger transcriptional factor Slug transcriptionally downregulates ERα by recruiting lysine-specific demethylase 1 in human breast cancer

**DOI:** 10.1038/oncsis.2017.38

**Published:** 2017-05-08

**Authors:** J-W Bai, M-N Chen, X-L Wei, Y-Ch Li, H-Y Lin, M Chen, J-W Li, C-W Du, K Man, G-J Zhang

**Affiliations:** 1 The Breast Center, Cancer Hospital of Shantou University Medical College (SUMC), Shantou, China; 2 ChangJiang Scholar’s Laboratory of SUMC, Shantou, China; 3 Department of Pathology, Cancer Hospital of SUMC, Shantou, China; 4 Department of Breast and Thyroid Surgery, The First Affiliated Hospital of SUMC, Shantou, China; 5 Department of Breast Medical Oncology, Cancer Hospital of SUMC, Shantou, China; 6 Department of Surgery, HongKong University Li Ka-Tsing Faculty of Medicine, Hongkong, China

## Abstract

Estrogen receptor α (ERα) is related with epithelial–mesenchymal transition, invasion and metastasis, and serves as an important therapeutic predictor and prognostic factor in breast cancer patients. The triple negative breast cancer (TNBC) is characterized by loss of hormone receptors and human epidermal growth factor receptor 2 (Her2), and lacks effective targeted therapy with poor prognosis. Unfortunately, the molecular mechanisms of ERα deficiency, which becomes hormone independent and results in resistance to endocrine therapy, remain to be elucidated in breast cancer. In this study, we observed an inverse correlation between Slug, a zinc-finger transcriptional repressor, and ERα expression in both human breast cancer tissues and cell lines. In ERα-negative breast cancer patients, high Slug messenger RNA expression showed obviously shorter relapse-free survival. We found that Slug binds to the E-box located in the promoter of estrogen receptor 1 gene (*ESR1*) to suppress its expression. More specifically, Slug recruits lysine-specific demethylase 1 (LSD1) to the E-box and thereby inhibits ERα expression by demethylating H3K4me2, which is evidenced by the interaction between Slug and LSD1. Moreover, the amount of H3K4me2 binding to the E-box was significantly increased after LSD1 knockdown in MDA-MB-231 cells. Functionally, the ability to proliferate, invade and metastasize was significantly suppressed after knockdown of either Slug or LSD1 alone, or both simultaneously. Taken together, these results suggest that Slug transcriptionally inhibits ERα expression by recruiting LSD1 to the ESR1 promoter in breast cancers. Thus, targeted inhibition of Slug and LSD1 may restore ERα and lead to resensitization to hormone therapy, providing a novel therapeutic strategy for ERα-negative breast cancer patients, especially for TNBC.

## Introduction

Breast cancer is a heterogeneous disease.^1^ With the development of DNA microarray technology, breast cancers were divided into four subtypes: estrogen receptor (ER)+/luminal-like, basal-like, Erb-B2+ and normal breast-like.^2^ According to intrinsic properties and different outcomes of the tumors, ER+/luminal-like group was further classified into at least two subgroups: luminal A and luminal B+C subtypes.^3^ Different therapeutic strategies are applied according to the molecular features of each subtype. For example, the majority of ER+/luminal-like tumors that are positive for ER and/or progestrone receptor (PR) respond well to hormonal interventions, whereas Erb-B2+ tumors characterized by amplified *ERBB2* oncogene and/or Her2 protein overexpression can be effectively controlled by using various anti-Her2 therapies.^4^ Therefore, breast cancer patients have greatly benefited from molecular classification that is used for individualized therapies and results in obvious improvements in disease-specific survival.^5^

Unfortunately, triple negative breast cancers (TNBCs) do not have effective targeted agents due to a lack of hormone receptors and Her2 expression. Developing novel therapeutic strategies has become paramount and imminent for patients with TNBC, especially in the era of individualized medicine.

ERα plays an important role in regulating mammary epithelial cell proliferation, differentiation and tumorigenesis.^6^ ERα can both activate and repress the expression of downstream target genes as a ligand-activated transcription factor.^7^ ERα does also serve as an important prognostic factor in breast cancer,^8^ and luminal subtype A tumors have the best clinical outcome among all subtypes.^3^

Many groups have focused on understanding the correlation between ERα expression loss and epithelial–mesenchymal transition (EMT), invasion and metastasis in TNBC. ERα, as a biomarker for terminally differentiated non-cancer normal breast luminal cells,^9^ promotes the growth of primary breast cancers, but can also antagonize signaling pathways that lead to EMT.^10, 11, 12, 13, 14, 15^ Moreover, our recent study demonstrated that ERα can suppress EMT and cancer cell stemness by downregulating the core subunit of the PRC1 complex, Bmi-1.^16^ Another study revealed that reduced E-cadherin expression is significantly associated with negative ERα expression in non-lobular breast carcinomas.^17^ Obviously, ERα loss is closely related to more serious malignant behaviors and poorer survival in TNBC patients. Why and how, then, is ERα lost in breast cancers, especially in TNBC? Therefore, determining the mechanism of ERα loss in breast cancers, including TNBC, is of vital importance.

Slug, a member of the Snail family, is C2H2-type zinc-finger transcription factor. It plays an essential role in development and cancer-associated EMT. Slug protein binds to E-box motifs in the promoter of downstream genes, and is shown to repress E-cadherin transcription in breast carcinoma, gastric carcinoma and esophageal squamous cell carcinoma.^18, 19, 20^ Slug is also demonstrated to negatively relate to ERα in both breast cancer and lung cancer.^15, 21^ ERα appears to downregulate Slug expression by following these mechanisms: first, directly repressing Slug transcription by forming a complex of HDAC1 N-CoR and ligand-activated ERα that binds to Slug promoter;^11^ second, activating the PI3K/Akt/GSK-3β pathway, leading to GSK-3β phosphorylation, and subsequent ubiquitination and proteasomal degradation of Slug.^22^ On the other hand, it is previously reported that Slug might downregulate ERα expression by targeting miR-221 for its activation,^23^ or by directly binding to the E-boxes of ESR1 promoter for repression.^24^

LSD1, the first identified histone demethylase, exhibits distinct transcriptional activity by maintaining an unmethylated status of H3K4 or K9.^25^ LSD1 stimulates ERα and androgen receptor (AR)-dependent transcription by demethylation of histone H3K9.^26, 27^ LSD1 leads to gene repression by catalyzing demethylation of mono and dimethylated H3K4 when interacting with different co-repressor complexes, such as CtBP,^28^ the Co-Rest^29^ complex and a subset of HDAC complexes.^30, 31^ In ERα-negative breast cancers, LSD1 is highly expressed and predicts an aggressive biological behavior.^32^ Most importantly, Slug could recruit LSD1 to the promoter of a few target genes for transcriptional repression.^33^ These findings prompt us to investigate whether Slug could cooperatively suppress ERα via forming complex with LSD1 in breast cancers.

In this study, we demonstrate a novel mechanism that the zinc-finger transcription factor Slug suppresses ERα expression via forming a complex with LSD1. Slug first binds to the E-boxes in the ESR1 promoter and further recruits epigenetic modifier LSD1 to inhibit ERα transcription in the human TNBC.

## Results

### Slug expression is negatively associated with ERα status in breast cancer patients

To explore the association between Slug and ERα, we selected 50 breast cancer patients in the Breast Center of the Cancer Hospital of Shantou University Medical College. We confirmed that both ERα and Slug were localized to nuclei (Supplementary Figure 1A) via immunohistochemical (IHC) staining analysis. Out of 50 patients, 36 were ERα positive (72%) and 14 were ERα negative (14/50; 28%). As compared to that of ERα-negative patients, Slug expression was much lower in ERα-positive patients (Table 1a; *P*=0.013).

**Figure 1:**
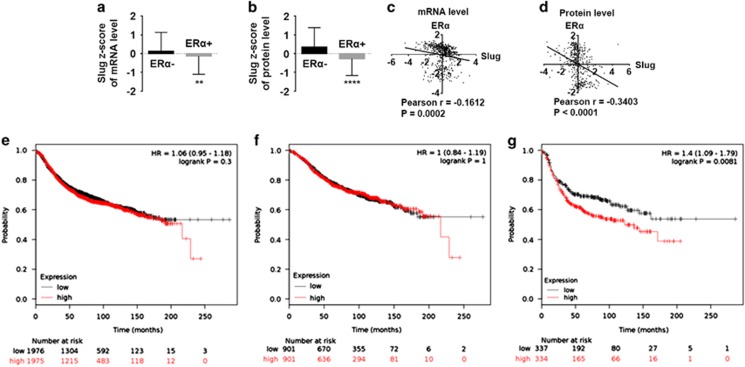
Negative correlation between Slug and ERα expression exists in human breast cancer tissues, and high Slug mRNA expression is correlated to poorer RFS in ERα-negative breast cancer patients. (**a**,** b**) Correlation of Slug mRNA (**a**) and protein (**b**) levels in ERα-negative and -positive breast cancer patients with *z*-score analysis. (**c**, **d**) Slug and ERα correlation analysis in *z*-scores of mRNA level (**c**) and protein level (**d**) is performed by two-tailed Pearson’s *R* tests. *Z*-scores of Slug and ERα are directly obtained from the online data (www.cbioportal.org). The formula is: *z*=(expression in tumor sample−mean expression in reference sample)/s.d. of expression in reference sample. (**e**–**g**) The prognostic effect of Slug mRNA is obtained from the website: www.kmplot.com. Survival curves are plotted for all patients (*n*=3951) (**e**) for ERα-positive cancers (*n*=1802) (**f**) and for ERα-negative cases (*n*=671) (**g**). Statistical analysis is performed using GraphPad Prism version 7.0 (GraphPad Software Inc., San Diego, CA, USA). *P<0.05, **P<0.01, ***P<0.001 and ****P<0.0001 (Student’s *t*-test) as compared to the control group. HR, hazard ratio.

To validate our results, we analyzed 825 breast cancer samples from The Cancer Genome Atlas (TCGA) database (2012, Nature); 528 cases among those patients had complete messenger RNA (mRNA) and 408 with protein level *z*-scores. We studied the *z*-scores of Slug mRNA and protein levels in both ERα-positive and ERα-negative patients, finding that both Slug mRNA and protein levels in ERα-positive patients were much lower than those of ERα-negative patients (Figures 1a and b). Moreover, Slug expression was negatively correlated with ERα at both mRNA level (*P*=0.0002, Figure 1c) and protein level (*P*<0.0001, Figure 1d). We also found that Slug protein level positively correlate with TP53 (*P*<0.0001, Supplementary Figure 1B), N-cadherin (*P*=0.0003, Supplementary Figure 1C) and negatively correlate with PR (*P*<0.0001, Supplementary Figure 1E), but no correlation with E-cadherin (*P*=0.0828; Supplementary Figure 1D). In summary, these data indicate an inverse correlation between Slug and ERα expression in breast cancer tissues.

### Slug is highly expressed in basal-like breast cancers, and predicts poor RFS

The relationship between protein expression data and clinic-pathological data in TCGA (2012, Nature) were analyzed from 408 breast cancer samples. We found that Slug had no obvious relationship with age, tumor size, lymph node status, American Joint Committee On Cancer (AJCC) stage, metastatic status or Her2 status. However, we found that Slug had a strong relationship with ER, PR status and molecular subtype. The Slug high-expression group had much higher percentage of basal-like breast cancer patients than the Slug low-expression subgroup (32.4–11.6% *P*<0.0001; Table 2).

We next examined the prognostic effect of Slug expression to breast cancer patients in the website www.kmplot.com. Survival curves were generated for all breast cancer cases (*n*=3951; Figure 1e), ERα-positive patients (*n*=1802; Figure 1f) and for ERα-negative patients (*n*=671; Figure 1g). After following up for 20 years, no association was found between Slug mRNA expression and relapse-free survival (RFS) for all breast cancers (*P*=0.3) or ERα-positive patients (*P*=1). However, high Slug mRNA expression was significantly associated with shorter RFS in ERα-negative patients (*P*=0.0081).

### Slug expression negatively correlates with ERα in breast cancer cells

**Figure 2:**
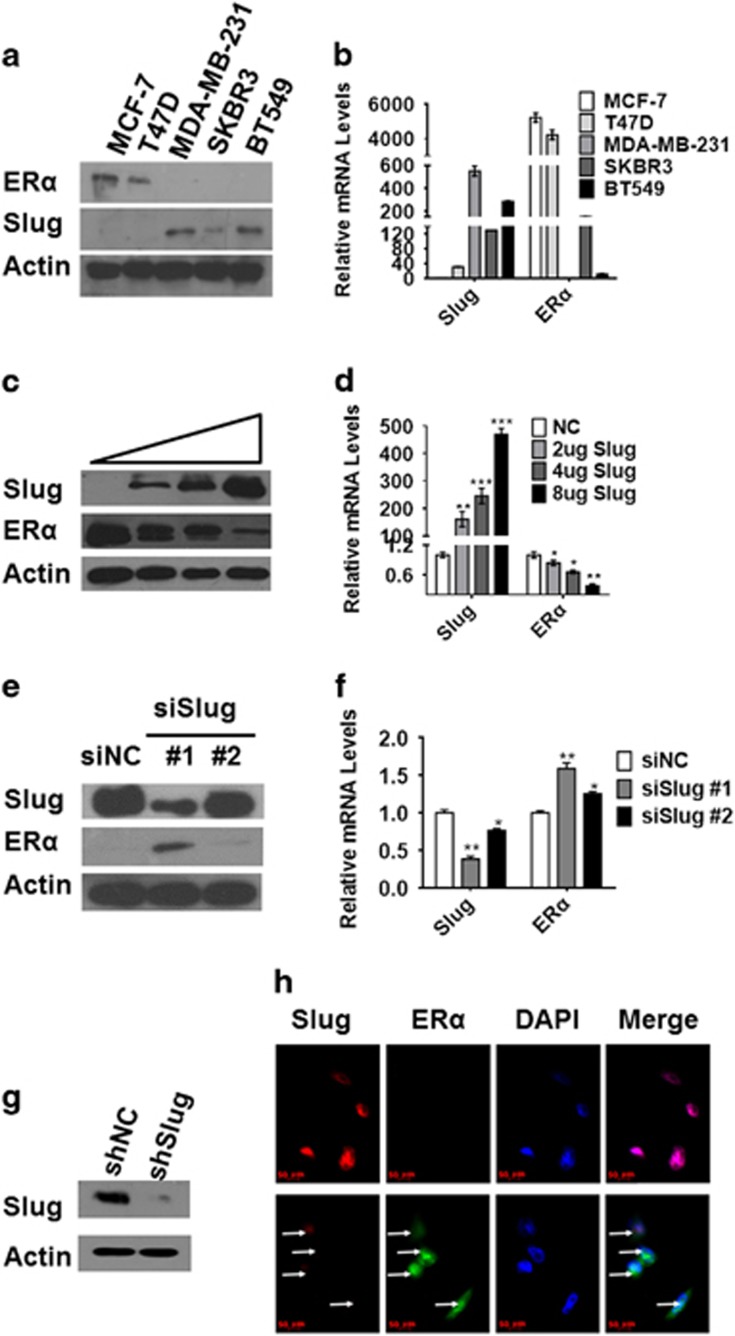
Negative relationship between Slug and ERα in human breast cancer cells. (**a**,** b**) Analysis of Slug and ERα expression levels in MCF-7, T47D, SKBR3, MDA-MB-231 and BT549 by western blot and RT–PCR. (**c**, **d**) Western blot (**c**) and RT–PCR (**d**) analysis of protein and mRNA levels as indicated in MCF-7 cells transfected with increasing concentrations (2, 4 and 8 μg, respectively) of Slug. (**e**,** f**) Western blot (**e**) and RT–PCR (**f**) analysis of protein and mRNA levels in MDA-MB-231 cells transfected with two different interference sequences of Slug (40 nm, separately). (**g**) Western blot analysis of Slug knockdown level in stable cell lines of MDA-MB231shSlug. (**h**) Immunofluorescence micrographs of Slug (red) and ERα (green) expression in MDA-MB-231shNC cells (upper) and MDA-MB-231shSlug (lower). DAPI is for nuclei (blue). Magnification, × 400. Protein and mRNA expression was normalized to β-actin. In figure 2d, NC means transfecting with control pcDNA3.1. In figure 2e and f, siNC is scrambled control siRNA used for transient transfection. ShNC without shRNA is used as a control by infecting pLKO.1 lentivirus in MDA-MB-231 cell line. Statistical analysis is performed using GraphPad Prism version 7.0. Means±s.d. is calculated by at least three independent experiments. *P<0.05, **P<0.01, ***P<0.001 and ****P<0.0001 (Student’s *t*-test) as compared to the control group. RT–PCR, reverse transcription–PCR.

To explore whether this negative correlation in breast cancer patients holds true in breast cancer cell lines, Slug and ERα protein and mRNA expression levels were detected in five different breast cancer cell lines: MCF-7, T47D, MDA-MB-231, SKBR3 and BT549. We found that Slug protein was highly expressed in ERα-negative MDA-MB-231, SKBR3 and BT549 cell lines; however, we did not find Slug expression in ERα-positive MCF-7 and T47D cell lines (Figure 2a). To compare Slug mRNA expression in those cell lines above, real-time reverse transcription–PCR was performed with β-actin as an internal control. Consistent with Slug protein expression, Slug mRNA levels were >500 times higher in MDA-MB-231 than that in MCF-7 cells (Figure 2b).

As both protein and mRNA levels of Slug were lower in ERα-positive cell lines, we hypothesized that the transcriptional repressor Slug may impact on ERα expression. Thus, we overexpressed Slug in MCF-7 cells at increasing concentrations (2, 4 and 8 μg, respectively) and knocked down endogenous Slug with 40 nm small-interfering RNA (siRNA) in MDA-MB-231 with two different interference sequences. We found that ERα was repressed in MCF-7 cells at both the protein and mRNA levels, and the extent of reduction was dose-dependent when overexpressing Slug (Figures 2c and d). In MDA-MB-231 cells, we detected ERα protein expression correlated with the level of Slug knockdown (Figure 2e). Real-time PCR revealed 1.6-fold increase in ERα mRNA level with RNA interference sequence #1, which was more effective (Figure 2f). Thus, we selected this sequence in the subsequent experiments. We repeated the above experiments in T47D and BT549 cells, finding congruent results (Supplementary Figures 1F and G). When Slug was stably knocked down in MDA-MB-231 cells (Figure 2g), ERα staining increased over that of control cells (Figure 2h). When we overexpressed Slug in MCF-7 (Supplementary Figure 1H) or T47D (Supplementary Figure 1L) cells, ERα immunofluorescence staining faded. Taken together, these data demonstrate that negative correlation between Slug and ERα exists in breast cancer cell lines.

### Slug downregulates ERα expression by binding to the ESR1 promoter

**Figure 3:**
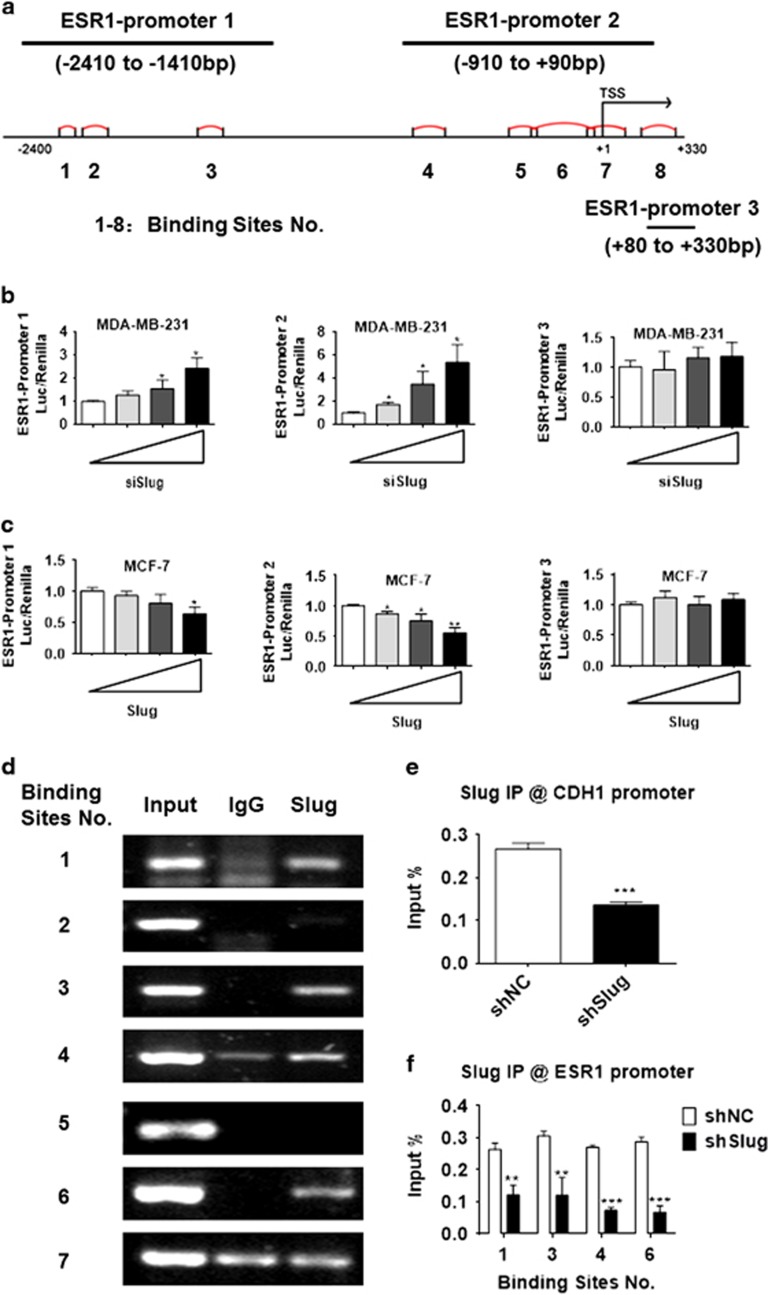
ERα expression is directly regulated by Slug. (**a**) A schematic representation of the ESR1 promoter region in which the promoter is divided into three subregions including eight E-box-binding sites. (**b**) Different concentration of Slug siRNA (20, 40 or 80 nm, respectively) was co-transfected with the ESR1 promoter-luciferase plasmid in MDA-MB-231 cells. Variation in transfection efficiency was normalized by Renilla luciferase activity. (**c**) Different concentration of Slug (0.1, 0.3 or 0.9 μg, respectively) was co-expressed with the ESR1 promoter-luciferase construct in MCF-7 cells. After 24 h, Renilla luciferase activity was used to normalize variation in transfection efficiency. (**d**) Normal IgG or anti-Slug antibodies were used in a ChIP assay to determine which E-box-binding site Slug binds in the ESR1 promoter in MDA-MB-231 cells. Seven primer pairs covering different binding sites within promoter 1 and 2 are designed. (**e**, **f**) After ChIP, qPCR was used to analyse Slug recruitment on CDH1 (**e**) and ESR1 (**f**) promoter in MDA-MB-231shSlug cells and their control cells. The results represent % of input chromatin. Statistical analysis is performed using GraphPad Prism version 7.0. Means±s.d. is calculated by at least three independent experiments. *P<0.05, **P<0.01, ***P<0.001 and ****P<0.0001 (Student’s *t*-test) as compared to the control group.

To determine whether Slug could bind to the ESR1 promoter, we generated three luciferase reporter vectors: ESR1 promoter 1-luc (−2410 bp to −1410 bp), ESR1 promoter 2-luc (−910 bp to +90 bp) and ESR1 promoter 3-luc (+80 bp to +330 bp), in which ESR1 promoter 1-luc covered first three putative binding sites, ESR1 promoter 2-luc covered the fourth through seventh binding sites and ESR1 promoter 3-luc covered the last binding site (Figure 3a). To functionally verify the involvement of Slug in ERα downregulation and elucidate which parts of ESR1 promoter were required, we conducted a dual-luciferase reporter assay in MDA-MB-231 and MCF-7 cells. Transfection with different concentrations (20, 40 or 80 nm, respectively) of siSlug significantly increased ESR1 promoter 1 and 2-luc activity in a dose-dependent manner, but no significant change at ESR1 promoter 3-luc was observed (Figure 3b). Similar results were found in MCF-7 and MCF-7Slug cells (Figure 3c).

To address whether Slug can bind to E-boxes in the ESR1 promoter, chromatin immunoprecipitation (ChIP) assays were carried out in MDA-MB-231 cells expressing high level of endogenous Slug. Anti-Slug antibody was used to identify the Slug/E-box-binding sites on the ESR1 promoter with non-specific IgG as a negative control. The results showed that Slug bound to the first, third, fourth and sixth binding sites, but not the second, fifth or seventh binding sites (Figure 3d). ChIP-quantitative PCR (qPCR) was then performed in MDA-MB-231shSlug cell lines that stably infected with lentivirus expressing Slug short hairpin RNA and control cells. The amounts of Slug binding to E-boxes of the CDH1 or ESR1 promoters in MDA-MB-231shSlug markedly decreased when compared with control cells (Figures 3e and f). These results reveal that Slug suppresses ERα transcription by binding to E-box motifs in the ESR1 promoter.

### The Slug/LSD1 complex regulates ERα expression in MDA-MB-231 cells

**Figure 4:**
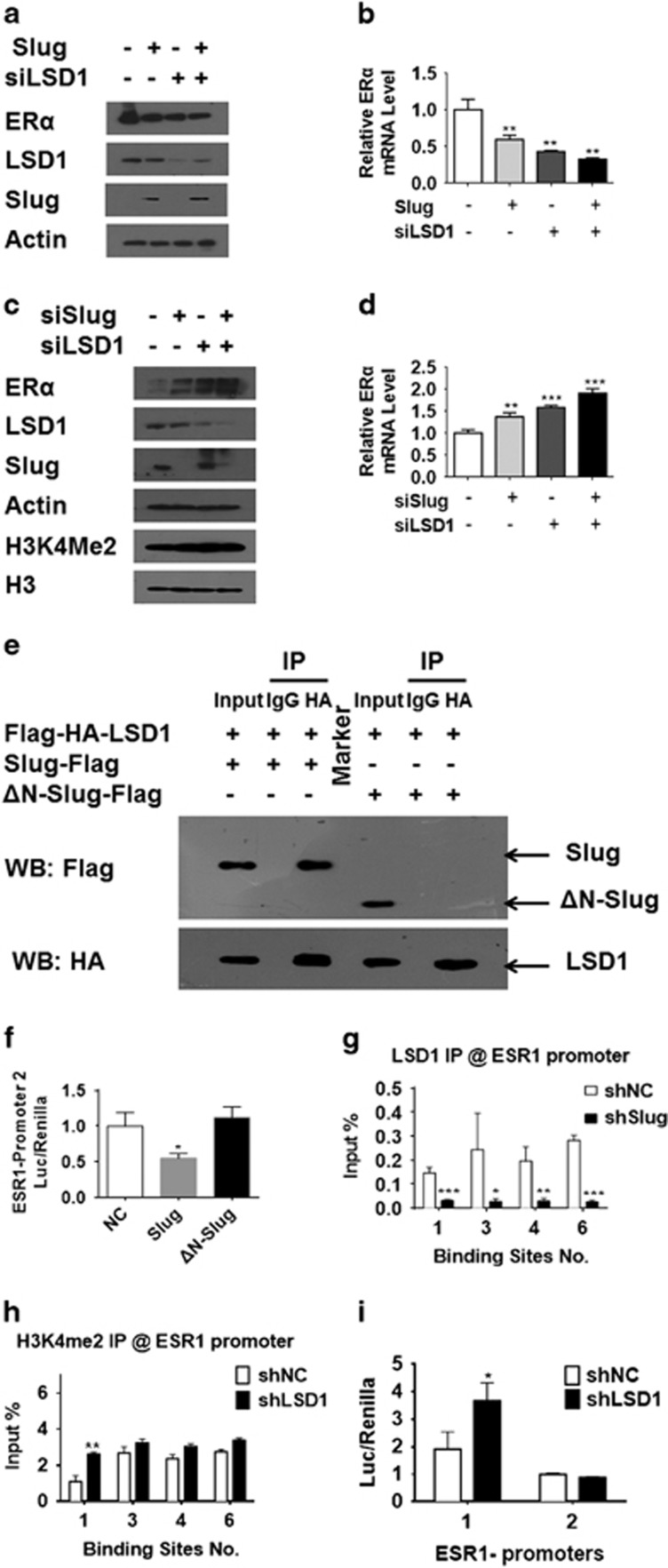
LSD1 acts cooperatively with Slug to inactivate ESR1 promoters by demethylating H3K4me2. (**a**, **b**) Western blot (**a**) and RT–PCR (**b**) analyze effects of LSD1 and Slug on ERα expression via Slug overexpression and LSD1 knockdown in MCF-7 cells. (**c**,** d**) Western blot (**c**) and RT–PCR (**d**) analysis of effects of LSD1 and Slug on ERα expression via Slug and LSD1 knockdown in MDA-MB-231 cells. (**e**) MDA-MB-231 cells expressing pHAGE-CMV-Flag-HA-LSD1 and pcDNA3.1-Slug-Flag or pcDNA3.1-ΔN-Slug-Flag was analyzed by IP. Cell lysates were immunoprecipitated with anti-HA antibody. Immunocomplexes were then immunoblotted using antibodies against Flag and HA. (**f**) A dual-luciferase reporter assay is used to investigate ESR1 promoter 2 activity in MDA-MB-231 cells by overexpressing wild-type or mutant Slug. (**g**) ChIP-qPCR analysis of LSD1 recruitment on ESR1 promoter in MDA-MB-231shSlug cells and their control cells. (**h**) ChIP-qPCR analysis of H3K4me2 recruitment on ESR1 promoter in MDA-MB-231shLSD1 cells and their control cells. All the ChIP-qPCR results represent % of input chromatin. (**i**) A dual-luciferase reporter assay is used to investigate ESR1 promoter activity in MDA-MB-231 cells after LSD1 knockdown. Statistical analysis is performed using GraphPad Prism version 7.0. Means±s.d. is calculated by at least three independent experiments. *P<0.05, **P<0.01, ***P<0.001 and ****P<0.0001 (Student’s *t*-test) as compared to the control group.

To explore if ERα expression is regulated by Slug/LSD1 complex in breast cancer cells, we first tested the effects of Slug and LSD1 on ERα expression in MCF-7 (Figures 4a and b) and MDA-MB-231 cells (Figures 4c and d). We found that knocking down LSD1 led to repression of ERα in MCF-7 cells. In contrast, ERα expression increased when LSD1 was knocked down in MDA-MB-231 cells. These data suggest Slug regulates ERα independent of LSD1 in MCF-7 cells. Thus, MDA-MB-231 cells were used as a study model to investigate the role of LSD1 in the regulation of ERα in the following experiments.

To study if the de-repression of ERα observed after Slug silencing was associated with LSD1, co-IP was conducted in MDA-MB-231 cell lines transiently co-transfected with HA-tagged LSD1 and Flag-tagged Slug. Anti-HA antibody was then used to isolate interacting proteins from whole-cell extracts. We found that Slug protein was immunoprecipitated with LSD1 (Figure 4e). While, a mutant form of Slug, ΔN-Slug, that lacks SNAG domain, failed to be immunoprecipitated by LSD1 (Figure 4e). These data verify that Slug specifically associates with LSD1, and the SNAG domain is necessary for this combination.

To look at the effect of ΔN-Slug on ESR1 promoter activity, we conducted a dual-luciferase reporter assay in MDA-MB-231 cells. As presented in Figure 4f, the activity of ESR1 promoter 2-luc was not affected by overexpressing ΔN-Slug compared with untransfected cells. We next endeavored to investigate if the ability of LSD1 to bind E-boxes was affected by Slug knockdown using a ChIP-qPCR assay in MDA-MB-231shNC and MDA-MB-231shSlug cells with anti-LSD1 antibody with the CDH1 promoter as a positive control (Supplementary Figure 2A). When Slug was knocked down, LSD1 binding to the ESR1 promoter significantly decreased (Figure 4g). These data suggest that Slug and LSD1 complexes bind to the ESR1 promoter.

To further determine the role of Slug/LSD1 complex, H3K4me2 levels were detected by western blot after Slug or LSD1 knockdown in MDA-MB-231 cells. Interestingly, we found that H3K4me2 levels increased in sync with ERα expression changes (Figure 4c). Furthermore, ChIP-qPCR was performed in MDA-MB-231shNC and MDA-MB-231shLSD1 stable cell lines (Supplementary Figure 2B) using anti-H3K4me2 antibody. As shown in Supplementary Figure 2C, CDH1 promoter was used as a positive control, and we found that LSD1 knockdown enhanced H3K4 dimethylation at the first binding site of ESR1 promoter (Figure 4h).

In a dual-luciferase reporter assay, when LSD1 was knocked down in MDA-MB-231 cells, ESR1 promoter 1 activity significantly increased (Figure 4i), but no significant change in activity of ESR1 promoter 2 was observed, which is consistent with ChIP-qPCR results. These results demonstrate that Slug regulates endogenous ERα by recruiting LSD1 to form a complex, leading to LSD1-mediated reduction of H3K4me2 levels to inhibit ERα expression in MDA-MB-231 cells.

### Slug, LSD1 knockdown repress cell proliferation, migration, invasion and colony formation

**Figure 5:**
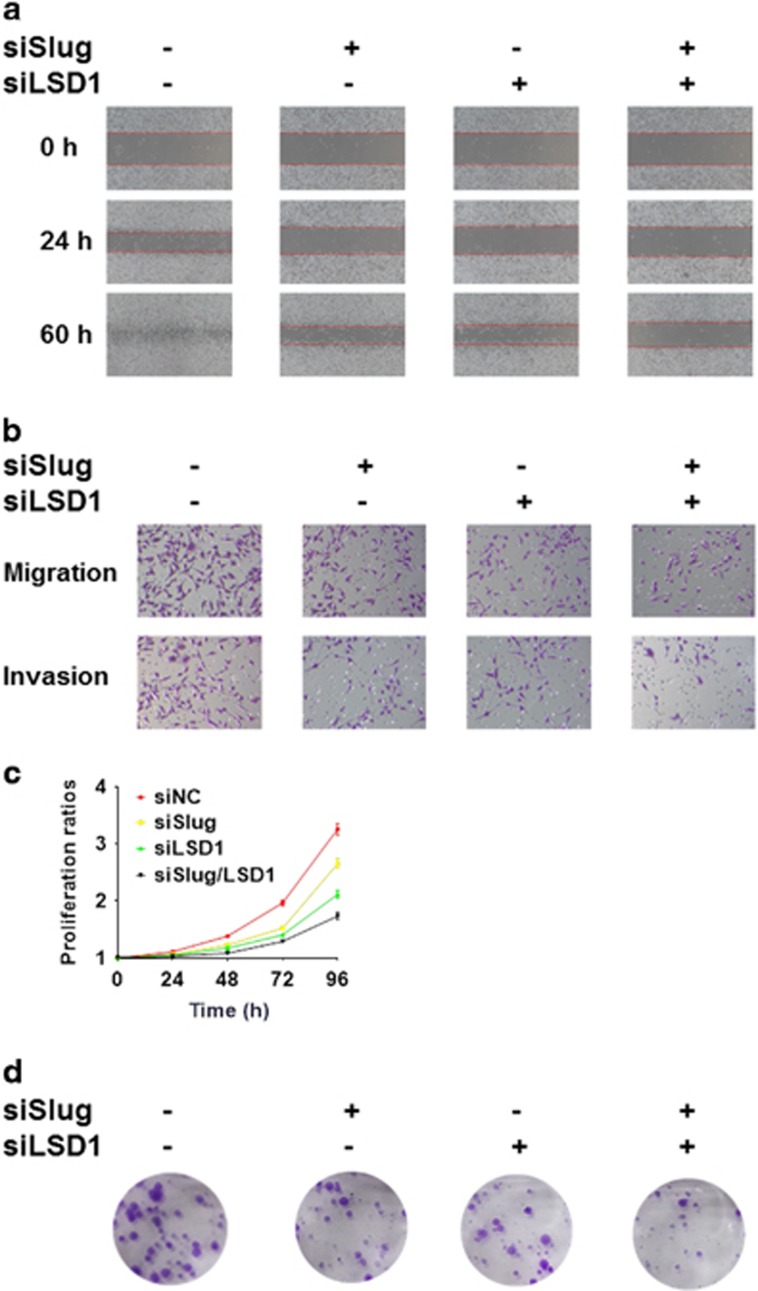
LSD1 acts cooperatively with Slug to promote proliferation, migration, invasion and colony formation abilities in MDA-MB-231 cell line. (**a**) A wound healing assay with the indicated siRNAs. Representative images are shown. Magnification, × 100. (**b**) Migration and invasion assays with the indicated siRNAs. Representative images of migrated (upper) and invaded (lower) cells are presented. Magnification, × 400. (**c**) Comparison of proliferative ability with the indicated siRNAs. (**d**) Colony formation ability with the indicated siRNAs. Statistical analysis is performed using GraphPad Prism version 7.0. Means±s.d. is calculated by at least three independent experiments. *P<0.05, **P<0.01, ***P<0.001 and ****P<0.0001 (Student’s *t*-test) as compared to the control group.

To assess the functional significance of Slug/LSD1 complex in tumorigenic and the proliferative ability, we performed wound healing assay, transwell assays, cell proliferation assay and colony formation assay in MDA-MB-231siSlug, MDA-MB-231siLSD1, and double knockdown MDA-MB-231siSlug+LSD1 cell lines as well as their control cell lines (Supplementary Figure 3A). The relative migrated distance at 24 h compared with their corresponding 0 h was about 38.5% in the control group, 23.1% in the MDA-MB-231siSlug group, 19.2% in the MDA-MB-231siLSD1 group and 7.7% in the double knockdown group. At 60 h, we could find similar results that healing of wounded areas in double knockdown group was lowest (Figure 5a, Supplementary Figures 3B and C). In the transwell assays, knockdown of Slug or LSD1 obviously decreased the level of migrated and invasive capacity, and especially double knockdown group markedly reduced by about 70% of migration and 74.3% of invasiveness compared to the control group (Figure 5b, Supplementary Figures 3D and E). In cell proliferation assay, cells transfected with siSlug or siLSD1 grew more slowly than control cells. Moreover, simultaneous Slug and LSD1 knockdown led to the lowest growth rate (Figure 5c). In the colony formation assay, colony formation ability decreased to 63.2% in Slug knockdown group, to 59.6% in LSD1 knockdown group and to 39.5% in double knockdown group in comparison with the control group (Figure 5d, Supplementary Figure 3F). These marked contrasts indicate that low expression of Slug or LSD1 inhibits breast cancer cell tumorigenic and proliferative ability.

## Discussion

**Figure 6:**
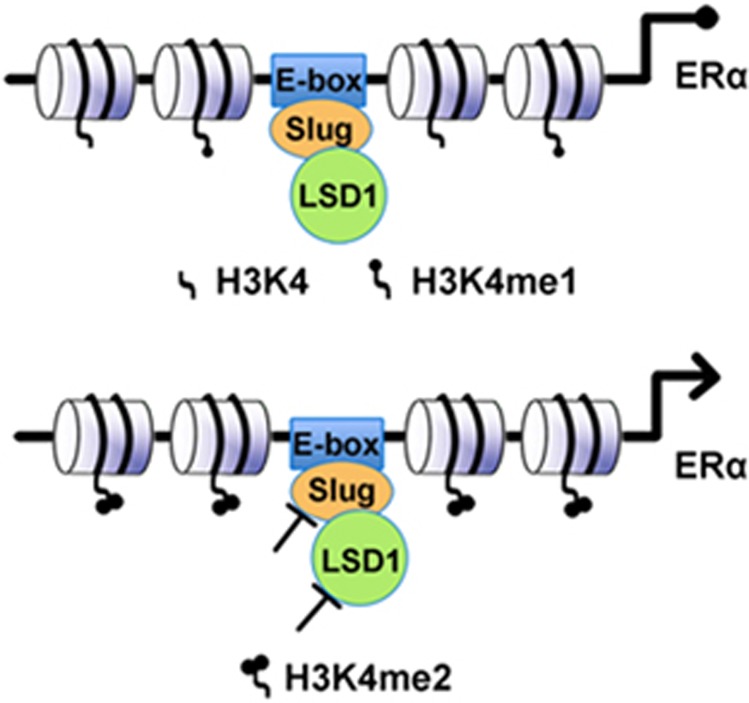
A schematic view of the importance of LSD1 recruitment by Slug for repressing ERα expression. Slug forms a complex with LSD1 and recruits LSD1 to E-boxes in the ESR1 promoter to suppress ERα transcription by demethylating H3K4me2. Inhibition of Slug or LSD1 or both result in decrease in demethylation of H3K4me2, leading to re-activation of ERα.

The molecular mechanism of ERα loss in breast cancers, especially in TNBC, has been extensively investigated. For example, it has been shown that many factors such as loss of heterozygosity, mutation in the ESR1 gene locus,^34, 35^ hypermethylation of CpG islands in the ESR1 promoter^36, 37^ or histone deacetylation of the *ESR1* gene^38, 39, 40^ contribute to ERα loss. In addition, miRNAs, including miRNA-18a^41^ and miR-22,^42^ miR-221/222, miR-206,^43, 44^ regulate ERα expression at transcriptional and post-transcriptional levels. It is noteworthy that Macaluso *et al.*^45^ demonstrated that an epigenetic repressor, the pRB2/p130-E2F4/5-histone deacetylase 1 (HDAC1)-DNA methyltransferase 1(DNMT1)-SUV39H1 complex, occupies the ESR1 promoter and regulates ERα transcription in ERα-negative MDA-MB-231 cell line. Subsequently, Vesuna *et al.*^46^ found that Twist recruits the HDAC1 and DNMT3B repressor complex to the ESR1 promoter, leading to repression of ERα expression and hormone resistance in breast cancer. In this study, we demonstrated a novel model of ERα repression by Slug based on epigenetic modification of the ESR1 promoter in breast cancer MDA-MB-231 cells, depicted in Figure 6.

First, we found that there exists a remarkable negative relationship between Slug and ERα in breast cancer cell lines and human breast cancer tissues. To demonstrate that our finding was not regional or incidental, we further analyzed their expression in breast cancer samples from a database generated as part of TCGA. The result showed that Slug indeed negatively correlated with ERα at both the mRNA and protein expression levels, suggesting the crucial role of Slug in regulating ERα expression. This result of negative correlation is also supported by the previous findings of Ye *et al*.^15^ In addition, high Slug expression was inversely associated with PR-positive expression, and was also found with high percentage in basal-like or called TNBC. However, Slug expression level was not associated with clinical parameters like patients’ age, tumor size and lymph nodes involved.

Furthermore, using an online database KM plot, we determined the prognostic significance of Slug in 3554 breast cancer patients. In ERα-negative breast cancer patients, high Slug mRNA expression showed significant shorter RFS. In contrast, no correlation was detected in ERα-positive breast cancer patients or total breast cancer patients regardless of ERα expression level. One possible reason for this result is that the percentage of ERα-positive breast cancer patients is relatively higher, confounding the prognostic value of Slug in ERα-negative breast cancer patients. Another reason is that Slug mRNA may not always correlate with its protein expression, making its effect on prognosis seemingly weak. Our combined results show that Slug is not only negatively associated with ERα in breast cancer tissues, but also predicts poor prognosis for ERα-negative breast cancer patients. This implies that Slug may accurately predict prognosis and even therapeutic response in ERα-negative patients. The zinc-finger domain near the C-terminus of Slug binds to specific DNA sequences, the E-box motif (5′-CANNTG-3′), while its N-terminus has a SNAG domain that mediates transcriptional repression.^47^ For example, Slug represses E-cadherin expression through binding to its E-box motifs in breast carcinoma, resulting in the induction of EMT.^48^ In this study, we divided ESR1 promoter containing eight potential Slug-binding sites into three regions. Quite intriguingly, we found that Slug can directly bind to the ESR1 promoter 1 and 2, but does not bind to ESR1 promoter 3 in either MDA-MB-231 or MCF-7 cells. Therefore, a series of primer pairs covering E-boxes in ESR1 promoter 1 and 2 were used for subsequent ChIP and ChIP-qPCR assays. We found that Slug could bind to at least four binding sites (1, 3, 4 and 6), but not to the remaining (2, 5 and 7). It may be necessary to perform more in-depth analysis to determine which E-box is more efficient and which is less efficient for Slug’s binding in the future.

LSD1 has been shown to repress the expression of BRCA1^49^ and E-cadherin^50^ through demethylation of H3K4. In an attempt to look at whether Slug cooperatively functions with LSD1, we found that ERα was much more greatly increased after Slug and LSD1 were knocked down simultaneously than that separately in the TNBC cell line MDA-MB-231, suggesting the cooperative action of two proteins. Co-IP studies revealed that Slug is associated with LSD1 when Slug and LSD1 were both overexpressed. In contrast, the mutant Slug (ΔN-Slug) lacking SNAG domain did not bind to LSD1, suggesting Slug’s specific association with LSD1 through its SNAG domain.

Furthermore, ChIP-qPCR with anti-LSD1 antibody in MDA-MB-231 cells revealed significant decrease in the Slug/LSD1 complex to E-boxes of ESR1 promoters when Slug was knocked down. ChIP-qPCR with anti-H3K4me2 antibody demonstrated an increased binding ability of H3K4me2 to E-boxes of ESR1 promoter 1 after knocking down LSD1 as compared to ESR1 promoter 2. Taken together, the above data suggest that Slug might recruit LSD1 to E-box motifs in the ESR1 promoter, and Slug/LSD1 complex demethylase H3K4me2 mainly through binding to the ESR1 promoter 1 region.

Moreover, we investigated whether LSD1 affects the activity of ESR1 promoter in MDA-MB-231 cells. ESR1 promoter 1 activity increased after LSD1 knockdown as compared to control, whereas the activity of ESR1 promoter 2 did not increase. This was consistent with our ChIP-qPCR results. Thus, the Slug/LSD1 complex is more effective in binding to the promoter region covering ESR1 promoter 1. Future studies are aimed at determining which E-boxes covered by ESR1 promoter 1 are most effective. Collectively, this study identifies that Slug’s binding to the E-boxes within the ESR1 promoter 1 is sufficient for ERα inhibition, and is also necessary for LSD1 recruitment in MDA-MB-231 cells.

Our recent study has demonstrated that ERα can suppress EMT, cell migration, invasion, metastasis, stemness,^16^ as well as reported by other groups.^15^ As expected, functional studies showed that cell proliferation, migration and invasion decreased, and these abilities were sharply weakened in MDA-MB-231 cells after simultaneous Slug and LSD1 knockdown. Thus, if one could re-activate ERα expression in ERα-negative breast cancer patients, especially in TNBC (for example, due to an epigenetic event or transcriptional repression), it may be reasonable to inhibit cancer progression and metastasis, and to resensitize to endocrine therapy or even chemotherapy. Given that Slug and LSD1 are upstream molecules that regulate of ERα expression, then these malignant biological behaviors might be brought under control by simultaneous Slug and LSD1 knockdown or their inhibition with small molecules.

Previously, both Snail and Slug have been shown to inhibit ERα expression through transcriptional repression.^24, 51^ Here, we report a different mechanism for Slug to repress ERα expression through recruiting LSD1 to the E-boxes of ESR1 promoter and demethylating H3K4me2 in ERα-negative breast cancers. Our findings, for the first time, illustrate the function of LSD1 in association with Slug to suppress ERα. Thus, our current results are conceptually in accordance with previous studies, but provide a novel mechanism responsible for Slug to repress ERα expression.

Ultimately, our study shows that Slug and LSD1 may be novel markers for aggressive biological behavior in ERα-negative breast cancer. The restoration of ERα may resensitize hormone resistance to endocrine therapy and improve clinical outcomes in ERα-negative breast cancer patients. Thus, targeted inhibition of the Slug/LSD1 complex may establish a highly specific method to restore ERα and to resensitize hormone therapy.

## Materials and methods

### Tissue specimens and immunohistochemistry

Fifty patients who underwent breast cancer surgery at the Cancer Hospital of Shantou University Medical College from March 2010 to May 2010 were selected to explore the relationship of Slug and ERα. Informed consent was acquired from all patients, and this study was approved by institutional ethics committee of the cancer hospital, Shantou University Medical College.

Immunohistochemistry (IHC) was performed as previously described^16^ using anti-Slug (Bioss Inc., Woburn, MA, USA) or an ERα antibody (Santa Cruz Biotechnology, Dallas, CA, USA) diluted 1:250 and 1:200, respectively. We used IHC scores according to the German Immunoreactive Scoring System^52^ to calculate Slug protein expression. The percentage of stained tumor cells was counted as follows 0 (none); 1(<10%); 2 (10–50%) and 3 (>50%). Staining intensity was calculated as follows: 0 (no staining); 1 (light yellow); 2 (yellow) and 3 (brown). The product of percentage × intensity was classified into low (⩽4) or high expression (>4).^53, 54^ ERα was scored as negative (−) if nuclear staining was showed in <10% of tumor cells. Judgment of IHC results was evaluated by two independent pathologists. Slides were photographed using a ZEISS Observer A1 inverted microscope (× 40 objective) (Carl Zeiss, Oberkochen, Germany).

### Online data acquisition and analysis

We obtained TCGA gene expression data from the website: www.cbioportal.org.^55, 56^ Clinical data were also directly obtained from this website. RNA sequencing data for Slug and ERα mRNA expression (*z*-score) were available for 528 of 825 breast cancer patients (2012, Nature), and reverse-phase protein array data for Slug and ERα protein expression (*z*-score) could be obtained for 408 breast cancer cases. *Z*-score for a sample means s.d. far away from the mean of referential expression. The formula is expressed as: *z*=(expression in tumor sample−mean expression in reference sample)/s.d. of expression in reference sample.

We used an online database and the website is as followed http://kmplot.com/analysis/index.php?p=service&cancer=breast to determine the relevance of Slug mRNA expression for RFS. The survival information and gene expression data are from >3000 breast cancer patients collected from Gene Expression Omnibus. The Affymetrix ID is valid: 213139_at (SNAI2). We set the cutoff point of Slug mRNA expression to 50%. Briefly, SNAI2 was uploaded into the database to get Kaplan–Meier survival plots with hazard ratio, the number-at-risk, 95% confidence intervals and the log-rank *P*-value that were counted automatically by the webpage.

### Cell culture and transfection

Human breast cancer cell lines BT549, MDA-MB-231, SKBR3, T47D, MCF-7, as well as HEK293 cells were purchased from American Type Culture Collection (ATCC, Manassas, VA, USA). Culture of all these cells followed manufacturer’s instructions.

Slug and LSD1 knockdown lentiviral production was generated by transfecting siSlug3 (plasmid # 10905 from Addgene, Cambridge, MA, USA) and shLSD1-pLKO.1 plasmids into HEK293 cells. In a sterile tube, siSlug3 or shLSD1-pLKO.1 was diluted in serum-free DMEM with viral packaging (psPAX2) and viral envelope (pMD2G) constructs at a 1:4:2 ratio. Polyethylenimine (1 μg/μl; Polysciences, Warrington, PA, USA) was mixed with the diluted DNA at a 3:1 ratio. The mixture of DNA/polyethylenimine was added to the cells after incubating for 20 min. Then we collected viral supernatant at 48 h after transfection. It was filtered and concentrated by ultracentrifugation. The aliquot virus was stored at −80 °C. It was used to infect MDA-MB-231 cell line supplemented with 8 μg/ml polybrene (Sigma-Aldrich, St Louis, MO, USA). After 48 h post infection, we began to select stable cell line by adding 2 μg/ml puromycin (Invitrogen Corp., Carlsbad, CA, USA) and then sustained it in a medium containing 500 ng/ml puromycin.

SiRNA transient transfection was carried out by means of Lipofectamine 2000 (Invitrogen Corp.). Briefly, Slug, LSD1 or scrambled control siRNA complex with transfection reagent was added into cultured MDA-MB-231 cells at 50% confluence. After 48 h, RNA or protein was extracted to detect transfection efficiency. We transfected 4 μg (scaling up or down transfections based on plating medium volume) pcDNA3.1-Slug-Flag or pcDNA3.1 into MCF-7 or T47D cells growing in 60 mm tissue culture plates using Lipofectamine 2000 for 48 h. Stably transfected Slug MCF-7 cells or T47D were selected in DMEM with 10% FBS plus 1 mg/ml G418 (Thermo Fisher Scientific, Waltham, MA, USA). After selection, stably transfected cells were cultured in medium with 500 μg/ml G418.

### Plasmids, siRNA and antibodies

siSlug3 (Addgene plasmid # 10905) was a gift from Dr Bob Weinberg. This plasmid was used to construct lentiviral vectors and the resulting stable cell line, MDA-MB-231shSlug. pHAGE-CMV-Flag-HA-LSD1 plasmid was kindly provided by Dr Yang Shi. Lentiviral short hairpin RNA pLKO.1 vectors targeting human LSD1 (shLSD1) were generated by inserting target sequence 5′-CCACGAGTCAAACCTTTATTT-3′, which was acquired by searching the pLKO.1-based TRC (the RNAi consortium) library. CDH1 promoter reporter was a kind gift from Dr Jianrong Lu.

A series of luciferase reporters driven by ESR1 promoter was constructed by inserting different ESR1 promoter sequences in pGL3-enhancer vector (Promega Corp., Madison, WI, USA). For example, ESR1 promoter 1-luc sequences included −2410 to −1410 bp, ESR1 promoter 2-luc included −910 to +90 bp and ESR1 promoter 3-luc included +80 to +330 bp. All promoter fragments were amplified by PCR.

Full length Slug complementary DNA was amplified from MDA-MB-231 cell line and cloned into pcDNA3.1-Flag (Promega Corp.). ΔN-Slug lacked the SNAG domain, and was cloned into pcDNA3.1-Flag.

Slug and scrambled siRNA was synthesized by Genepharma Biotech (GenePharma, Shanghai, China). Slug siRNA-1 sequences targeted 5′-GGACCACAGUGGCUCAGAA-3′ siRNA-2 sequences targeted 5′-GCATTTGCAGACAGGTCAAAT-3′. Scrambled siRNA sequences were 5′-UUCUCCCGAACGUUCACGU-3′ (sense) and 5′-ACGUGACACGUUCGGAGAA-3′ (antisense). LSD1 siRNA was obtained from Santa Cruz Biotechnology. The detailed information about antibodies used in this study was shown in Table 3.

### Immunofluorescence

MCF-7, T47D, MDA-MB-231, and Slug overexpressing or knockdown cell lines MCF-7Slug, T47DSlug and MDA-MB-231shSlug were cultured in Millicell EZ SLIDE 8-well glass (Merck Millipore, Darmstadt, Germany) to 80% confluence. After removing the medium and washing once with PBS, cells were fixed via 4% paraformaldehyde. Then cell membranes were permeated with 0.5% Triton-X-100 for 5–8 min. Heterogenetic antigens were blocked with 2.5% goat serum for 30 min, then cells were incubated with anti-Slug and anti-ERα antibody diluted 1:400 and 1:200, respectively, overnight at 4 °C. In the next day, after incubation with secondary antibody, cells were mounted in DAPI (Invitrogen Corp.) for 3–5 min. Slides were analyzed using a ZEISS Observer A1 inverted microscope (× 400 magnification) (Carl Zeiss).

### Luciferase reporter assay

We plated 1 × 10^5^ MDA-MB-231 cells in each well of 12-well plates. Cells were transfected with different concentration of siRNA against Slug (20, 40 or 80 nm) or 40 nm siRNA against LSD1, and 0.3 μg of the CDH1 promoter-luciferase plasmid or ESR1 promoter-luciferase plasmid per well. Cells were also co-transfected with 10 ng of pRL-SV40 (Renilla luciferase, Promega Corp.) to normalize transfection efficiency. Twenty-four hours after transfection, luciferase activity was measured by the Dual-Luciferase Assay kit (Promega Corp.). The relative luciferase was expressed as the ratio of firefly luciferase to Renilla luciferase.

To detect the effect of Slug on ESR1 promoter activities, we plated 2 × 10^5^ MCF-7 cells in each well of 12-well plates. Cells were transfected with 0.3 μg ESR1 promoter-luciferase plasmid along with 0.1, 0.3 or 0.9 μg of Slug and 10 ng of pRL-SV40 per well. Similar experiments were carried out to explore the effect of the mutant ΔN-Slug on the ESR1 promoter. Error bars indicate the s.d. from three independent experiments.

### Co-IP and ChIP

For immunoprecipitation, about 50% confluent MDA-MB-231 cells grown in a 100 mm dish were co-transfected with pHAGE-CMV-Flag-HA-LSD1 and pcDNA3.1-Slug-Flag or pcDNA3.1-ΔN-Slug-Flag plasmids (8 μg each) and then lysed in cell lysis buffer for western blot and IP (Beyotime Biotechnology, Shanghai, China) containing PMSF protease inhibitor (Beyotime Biotechnology). After preclearing, 1 μg cell lysates were incubated with 1 μg of rabbit-derived anti-HA or anti-IgG antibody for 2 h, and then 20 μl Protein A+G Plus Agarose was added into lysates with rotation overnight at 4 °C. Protein A+G beads were washed and immunoprecipitated complexes were resolved by 2 × electrophoresis sample buffer in the absence of 2-mercaptoethanol. Mouse-derived anti-Flag antibody was used for western blot to determine Slug or mutant Slug co-immunoprecipitation with LSD1.

For the ChIP assay, 2 × 10^7^ MDA-MB-231 cells were cross-linked with 1% formaldehyde and then stopped by 125 mm glycine. One milliliter of cold cell lysis/wash buffer (8.766 g NaCl, 0.5 M 10 ml EDTA pH 7.5, 50 ml 1 M Tris pH 7.5, 5 ml NP-40 and MilliQ H_2_O (Milli-Q System, Millipore, Billerica, MA, USA) up to 1 liter) containing protease inhibitor was then added. After sonication, samples were diluted in shearing buffer (50 ml 20% SDS, 20 ml 0.5 M EDTA pH 8.0, 50 ml 1 M Tris pH 8.0 and MilliQ H_2_O up to 1 liter), and then incubated with specific antibodies and Pierce Protein A/G Magnetic Beads (Invitrogen Corp.) overnight at 4 °C. To remove non-specific binding, immunoprecipitates underwent a series of wash steps. Through reverse cross-linking, purified DNA was resolved on a 1.5% agarose gel or analyzed by real-time qPCR, and represented by proportion of input chromatin. More than three independent experiments were performed to calculate means and s.d. The primers used in ChIP-qPCR are available in Supplementary Table 1.

### Cell proliferation assay

We seeded 4 × 10^3^ MDA-MB-231 cells with or without Slug or LSD1 knockdown in each well of 96-well microplate. Proliferation abilities were investigated by a Cell Counting Kit-8 (Beyotime Biotechnology). We measured absorbance of different groups at 450 nm with a microplate reader ELX800 (Bio-Tek, Winooski, VT, USA) from day 0 to day 4. Three independent experiments were carried out.

### Colony formation assay

We plated 200 MDA-MB-231 cells with or without Slug or LSD1 knockdown in each well of six-well plate. After 2 weeks, cells were fixed with methyl alcohol for 15 min and stained with Gentian Violet. Only colonies that had >50 individual cells were counted using a BX51 microscope (Olympus, Tokyo, Japan) (100 × magnification). The experiment was performed three times independently.

### Wound healing assay

MDA-MB-231 with or without Slug or LSD1 knockdown were plated in six-well plates at a 95% confluence. After starvation by depriving serum for 24 h, we created a linear wound with a pipette tip. Wound healing was photographed at 12 h intervals and the ratio of each scratch closure was obtained by comparing distance of scratch at X h with the corresponding one at time 0 h. The distance was measured by the software of BX51 microscope. The relative migration distance was calculated using the following formula: the relative migration distance (%)=100 (A_0 h_−_Ax h_)/A_0 h_. At least five readings of distance were measured for each sample and each experiment was repeated at least three times.

### Cell migration and invasion assay

Cell migration assays were conducted by using transwell chambers (Corning Life Sciences, NY, USA) as previously described.^57^ Briefly, transfected cells were starved for 24 h without serum, then 2 × 10^4^ cells were plated in the upper chamber. The lower chamber was added with DMEM plus 20% FBS. Forty-eight hours later, non-migrated cells from the upper side of the chamber were removed, and cells on the lower side of the chambers were fixed by methanol and stained with 0.1% crystal violet.

For cell invasion assays, 2 × 10^4^ MDA-MB-231 cells were seeded in the upper compartment of Matrigel-coated inserts (Corning Life Sciences). After 72 h, invaded cells were collected. The rest of the protocol was similar as cell migration assays. The mean number of the five fields represented migrated/invaded cells’ amount. All the fields were photographed with the use of a BX51 microscope (× 400 magnification). Each experiment was performed in triplicate independently.

### Statistical analysis

Differences between the distributions of categorical variables were evaluated by means of Pearson’s *χ*^2^-test. Correlations between Slug, LSD1 and ERα expression and other related factors were calculated using two-tailed Pearson’s *R* tests. Students’ *t*-test was carried out to assess the statistical differences between experimental and control groups. All the statistical analyses were performed by GraphPad Prism version 7.0. For all the analyses, *P*-value <0.05 was considered statistically significant.

## Figures and Tables

**Table 1:** Correlation between Slug and ERα expression detected by IHC in 50 breast cancer patients

*Slug expression*	*ERα expression*		P-*value*	R-*value*
	*Positive* n *(%)*	*Negative* n *(%)*		
High	8 (22.2%)	9 (64.3%)	0.013	−0.236
Low	28 (77.8%)	5 (35.7%)		

Abbreviations: ERα, estrogen receptor α IHC, immunohistochemistry.

Note: The Slug and ERα expression was measured by IHC and *P*-value was calculated using Pearson* χ*^2^-test. *R*-value indicated the correlation between Slug and ERα calculated by IHC quantitative scores using two-tailed Pearson’s *R* tests.

**Table 2:** Relationship between Slug expression and clinic-pathological parameters in breast cancer patients

Features	Slug expression (%)		χ^*2*^	P-*value*
	*Low (*n*=232)*	*High (*n*=176)*		
*Age*				
⩽50	68 (29.3)	54 (30.7)	0.090	0.76
>50	164 (70.7)	122 (69.3)		
				
*Tumor size*				
T1	53 (22.8)	39 (22.2)	1.87	0.6
T2	136 (58.6)	112 (63.6)		
T3	32 (13.8)	17 (9.7)		
T4	10 (4.3)	8 (4.5)		
N/A	1 (0.4)			
				
*Nodal status*				
N0	121 (52.2)	79 (44.9)	2.23	0.53
N1	70 (30.2)	61 (34.7)		
N2	27 (11.6)	25 (14.2)		
N3	14 (6.0)	11 (6.3)		
				
*AJCC stage*				
I	40 (17.2)	27 (15.3)	0.71	0.87
II	128 (55.2)	102 (58.0)		
III	49 (21.1)	39 (22.2)		
IV	9 (3.9)	5 (2.8)		
N/A	6 (2.6)	3 (1.7)		
				
*Metastasis status*				
M0	222 (95.7)	172 (97.7)	0.85	0.36
M1	9 (3.9)	4 (2.3)		
No data	1 (0.4)			
				
*ER*				
Negative	30 (12.9)	68 (38.6)	34.24	<0.000
Positive	195 (84.1)	108 (61.4)		
N/A	7 (3.0)			
				
*PR*				
Negative	63 (27.2)	91 (51.7)	23.46	<0.000
Positive	162 (69.8)	85 (48.3)		
N/A	7 (3.0)			
				
*Her2*				
Negative	191 (82.3)	139 (79)	0.84	0.36
Positive	32 (13.8)	30 (17)		
N/A	9 (3.9)	7 (4)		
				
*Molecular subtype*				
Luminal A/B	177 (76.3)	88 (50)	33.98	<0.000
Her2-enriched	22 (9.5)	26 (14.8)		
Basal-like	27 (11.6)	57 (32.4)		
Normal-like	6 (2.6)	5 (2.8)		

Abbreviations: ERα, estrogen receptor α N/A, not available; TCGA, The Cancer Genome Atlas.

Note: 408 breast cancer samples with *z*-scores of protein level were from the TCGA database. The expression of Slug and ERα measured by reverse-phase protein array was directly obtained from the online data (www.cbioportal.org). *P*-value was calculated using Pearson *χ*^2^-test.

**Table 3:** Antibodies used in this study

*Antibody*	*Cat. no.*	*Company*	*Usage*
Slug	bs-1382R	Bioss Inc.	IHC
Slug	9585S	Cell Signaling Technologies	WB; IF; ChIP
ERα	sc-543	Santa Cruz Biotechnology	WB; IHC
ERα	sc-8005	Santa Cruz Biotechnology	IF
LSD1	2184S	Cell Signaling Technologies	WB; ChIP
H3K4me2	9725S	Cell Signaling Technologies	WB; ChIP
H3	sc-10809	Santa Cruz Biotechnology	WB
HA	sc-805	Santa Cruz Biotechnology	IP; WB
Flag	F3165	Sigma-Aldrich	WB
β-Actin	sc-47778	Santa Cruz Biotechnology	WB
Goat anti-mouse IgG-HRP	sc-2005	Santa Cruz Biotechnology	WB
Goat anti-rabbit IgG-HRP	sc-2301	Santa Cruz Biotechnology	WB; IHC
Alexa Fluor 595 Goat Anti-rabbit IgG (H+L)	A11012	Thermo Fisher Scientific	IF
Alexa Fluor 488 Goat Anti-mouse IgG (H+L)	A11001	Thermo Fisher Scientific	IF

Abbreviations: ChIP, chromatin immunoprecipitation; ERα, estrogen receptor α IHC, immunohistochemistry; IF, immunofluorescence; WB, western blot.
