# Supplementary material for: The zinc-finger transcriptional factor Slug transcriptionally downregulates ERα by recruiting lysine-specific demethylase 1 in human breast cancer
Source: Oncogenesis. 2017 May 8;6(5):e330–. doi: 10.1038/oncsis.2017.38 (PMC5523071; doi:10.1038/oncsis.2017.38)
Supplement: Supplementary Information [file oncsis201738x1.docx]

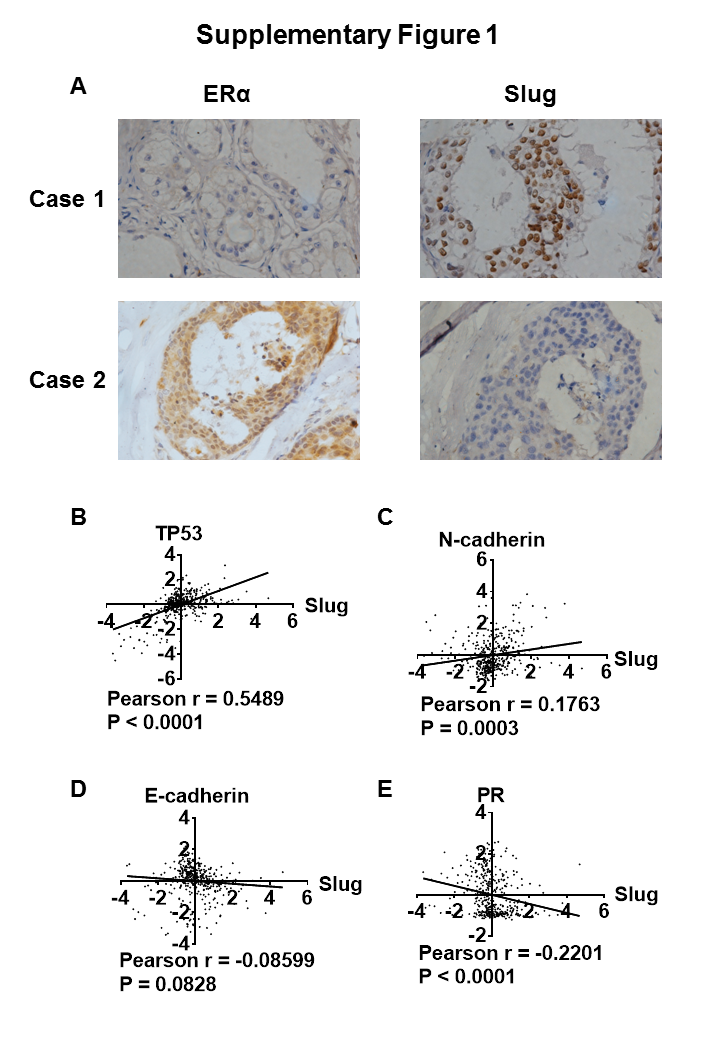

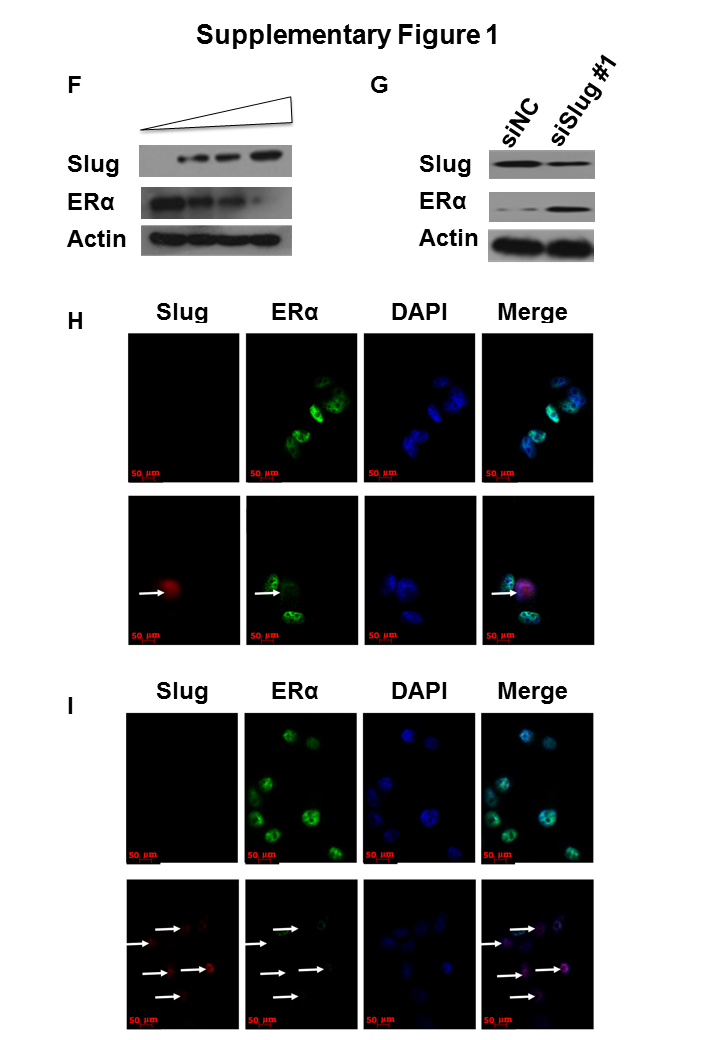

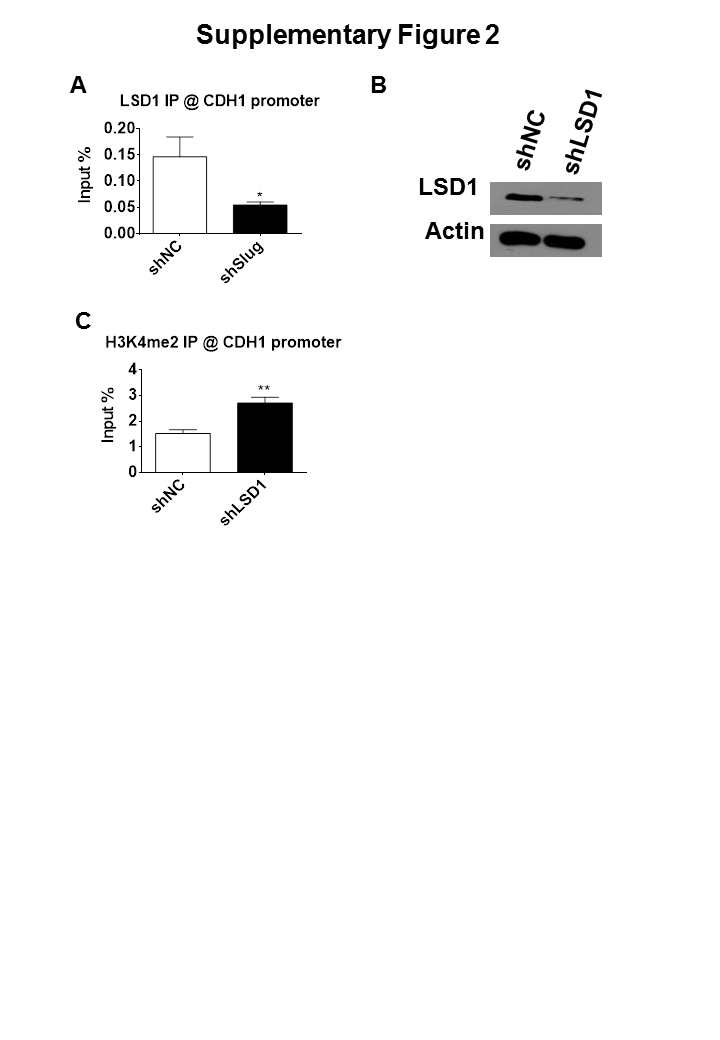

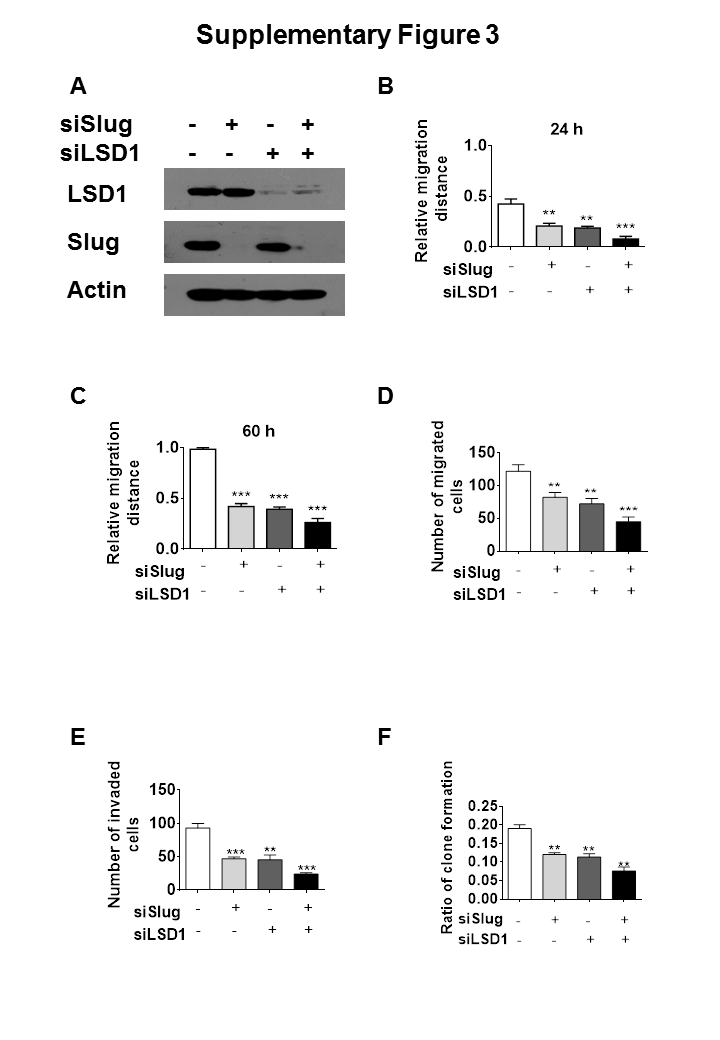


**Supplementary Figure 1: IHC staining of Slug and ERα in human breast cancer tissues and correlation between Slug and some EMT related factors protein expression in human breast cancer tissues.** (A) Immunohistochemistry micrographs of negative ERα, positive Slug in case 1 (upper row); and positive ERα, negative Slug in case 2 (lower row). Magnification, 400X (B-E) Protein expression correlation analysis of Slug and some EMT related factors are performed by two-tailed Pearson’s R tests: TP53 (B, Pearson’s R = 0.5489), N-cadherin (C, Pearson’s R = 0.1763), E-cadherin (D, Pearson’s R = -0.08599), PR (E, Pearson’s R = -0.2201). Negative correlation between Slug and ERα expression in human breast cancer cell lines. (F) Western blot analysis of ERα expression in MCF-7 cells transfected with increasing concentrations (2 µg, 4 µg, and 8 µg, respectively) of Slug. (G) Western blot analysis of ERα expression in BT549 cells knocked down Slug with 40nM interference sequence 1. (H & I) Immunofluorescence micrographs of Slug (red) and ERα (green) expression in Slug overexpressed cells MCF-7Slug (H lower), T47DSlug (I lower) and their control cells (H, I upper separately). DAPI is for nuclei (blue). Magnification, 400×. Statistical analysis was performed using GraphPad Prism version 7.0. * P < .05, ** P < .01, *** P < .001 and **** P < .0001.

**Supplementary Figure 2: LSD1 represses E-cadherin expression by binding to CDH1 promoter.** (A) ChIP-qPCR analysis of LSD1 recruitment on CDH1 promoter in MDA-MB-231shSlug cells and their control cells. (B) Western blot analysis of LSD1 knockdown level in stable cell lines MDA-MB231shLSD1. (C) ChIP-qPCR analysis of H3K4me2 recruitment on CDH1 promoter in MDA-MB-231shLSD1 cells and their control cells. All the ChIP-qPCR results represent percentage of input chromatin. Data are presented as the mean ± s.d. of three experiments. * P < .05, ** P < .01, *** P < .001 and **** P < .0001 (Student’s *t*-test) as compared to control cells.

**Supplementary Figure 3: LSD1 acts cooperatively with Slug to promote migration, invasion and EMT.** (A) Western blot analysis of interference effect of Slug and LSD1 in MDA-MB-231 cells which are used in subsequent experiments. (B & C) The statistical analysis of a wound healing assay in MDA-MB-231 cells with the indicated siRNAs at 24 h and 60 h separately. (D-F) The statistical analysis of migration (D) and invasion (E) assays and colony formation (F) assay in MDA-MB-231 cells with the indicated siRNAs. Data are presented as the means ± s.d. of at least three independent experiments. * P < .05, ** P < .01, ***P < .001 and ****P < .0001 (Student’s *t*-test) as compared to control cells.

Supplementary Table 1

| **Primers** | **Sequence** |
| --- | --- |
| **ChIP primers for ESR1 promoters** |  |
| Binding sites 1 Fwd | GTCTGGTTTCCTGGTGCAAT |
| Binding sites 1 Rev | CAAATGCCTTACTGGCCCTA |
| Binding sites 2 Fwd | CAGCAAGTCTCCCCTCACTC |
| Binding sites 2 Rev | ATTCGGGAAGCAGCCAGTAG |
| Binding sites 3 Fwd | TGAAGTGCTTTTTGCATGTG |
| Binding sites 3 Rev | CCTTTATGGCCAGCAATCAT |
| Binding sites 4 Fwd | TGGACCAGACCGACAATGTA |
| Binding sites 4 Rev | GTTCATGCCTTCCACAGGTT |
| Binding sites 5 Fwd | AGTTCCCCCAGCTGCTAAAT |
| Binding sites 5 Rev | ACCCTGGGGAGGACTACACT |
| Binding sites 6 Fwd | CAGCAGCGACGACAAGTAAA |
| Binding sites 6 Rev | TGCATTACAAAGGTGCTGGA |
| Binding sites 7 Fwd | ACAGTGTAGTCCTCCCCAGG |
| Binding sites 7 Rev | TTGTCGTCGCTGCTGGATAG |
| **ChIP primers for CDH1 promoters** |  |
| CDH1 promoter Fwd | GCTAGAGGGTCACCGCGTCTA |
| CDH1 promoter Rev | GCTTTGCAGTTCCGACGCCAC |
| **PCR primers** | Sequence |
| ERα Fwd | TGCTTCAGGCTACCATTATGGA |
| ERα Rev | TGGCTGGACACATATAGTCGTT |
| Slug Fwd | AAGCATTTCAACGCCTCCAAA |
| Slug Rev | GGATCTCTGGTTGTGGTATGACA |
| LSD1 Fwd | GTGCAGTACCTCAGCCCAAAG |
| LSD1 Rev | CCGAGCCCAGGGATCAG |
| β-actin Rev | AGCGAGCATCCCCCAAAGTT |
| β-actin Fwd | GGGCACGAAGGCTCATCATT |

Note: Fwd, forward; Rev, reverse
